# A tRNA splicing operon: Archease endows RtcB with dual GTP/ATP cofactor specificity and accelerates RNA ligation

**DOI:** 10.1093/nar/gkt1375

**Published:** 2014-01-16

**Authors:** Kevin K. Desai, Chin L. Cheng, Craig A. Bingman, George N. Phillips, Ronald T. Raines

**Affiliations:** ^1^Department of Biochemistry, University of Wisconsin–Madison, Madison, WI 53706, USA, ^2^Department of Biochemistry and Cell Biology and Department of Chemistry, Rice University, Houston, TX 77005, USA and ^3^Department of Chemistry, University of Wisconsin–Madison, Madison, WI 53706, USA

## Abstract

Archease is a 16-kDa protein that is conserved in all three domains of life. In diverse bacteria and archaea, the genes encoding Archease and the tRNA ligase RtcB are localized into an operon. Here we provide a rationale for this operon organization by showing that Archease and RtcB from *Pyrococcus horikoshii* function in tandem, with Archease altering the catalytic properties of the RNA ligase. RtcB catalyzes the GTP and Mn(II)-dependent joining of either 2′,3′-cyclic phosphate or 3′-phosphate termini to 5′-hydroxyl termini. We find that catalytic concentrations of Archease are sufficient to activate RtcB, and that Archease accelerates both the RNA 3′-P guanylylation and ligation steps. In addition, we show that Archease can alter the NTP specificity of RtcB such that ATP, dGTP or ITP is used efficiently. Moreover, RtcB variants that have inactivating substitutions in the guanine-binding pocket can be rescued by the addition of Archease. We also present a 1.4 Å-resolution crystal structure of *P. horikoshii* Archease that reveals a metal-binding site consisting of conserved carboxylates located at the protein tip. Substitution of the Archease metal-binding residues drastically reduced Archease-dependent activation of RtcB. Thus, evolution has sought to co-express *archease* and *rtcB* by creating a tRNA splicing operon.

## INTRODUCTION

Archease is a small (16 kDa) acidic protein that is conserved in eukarya, bacteria and archaea (1). The *archease* gene generally localizes adjacent to genes encoding enzymes and other proteins involved in DNA or RNA processing (1,2), including the tRNA ligase RtcB. The conservation of genomic context for *archease* suggests that Archease could function broadly as a modulator or chaperone of nucleic acid modifying proteins (1). In accord with a suspected role in assisting nucleic acid processing enzymes, Archease has been shown to increase the specificity of a tRNA m^5^C methyltransferase (2). The common organization of *rtcB* and *archease* into an operon suggests that Archease could also function to modulate the activity of RtcB. In a preliminary report, Martinez has put forth human Archease as an activator of human RtcB (3).

**Figure 1.**
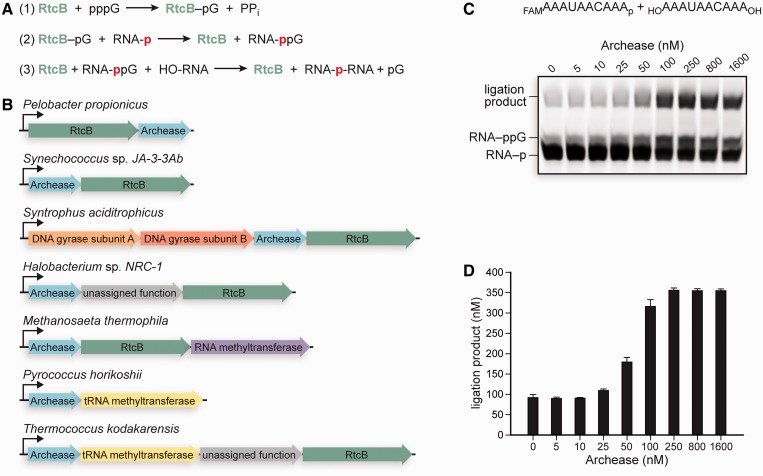
The three nucleotidyl transfer steps of catalysis by RtcB, a putative tRNA splicing operon and the titration of Archease into RNA ligation reactions with RtcB. (**A**) The three nucleotidyl transfer steps of catalysis by RtcB are (1) RtcB guanylylation, (2) RNA 3′-P guanylylation and (3) phosphodiester bond formation. (**B**) The operon organization of *rtcB* and *archease* in diverse bacteria (*Pelobacter propionicus*, *Synechococcus* sp. *JA-3-3Ab* and *Syntrophus aciditrophicus*) and archaea (*Halobacterium* sp. *NRC-1*, *Methanosaeta thermophila*, *P. horikoshii* and *Thermococcus kodakarensis*). (**C**) RtcB-catalyzed RNA ligation reactions titrated with increasing concentrations of Archease, as specified. Reaction mixtures contained 50 mM Bis–Tris buffer (pH 7.0), NaCl (300 mM), MnCl_2_ (0.25 mM), GTP (0.10 mM), *P. horikoshii* RtcB (5 μM), 5′ RNA fragment (1.0 μM) and 3′ RNA fragment (1.0 μM). (RNA substrates are shown at top.) Reaction mixtures were incubated at 70°C for 30 min, and quenched with an equal volume of RNA gel-loading buffer. The reaction products were resolved by electrophoresis through an 18% w/v urea–polyacrylamide gel and visualized by fluorescence scanning of the FAM label. (**D**) Ligation product (nM) plotted versus Archease concentration (nM). Values are the mean ± SE for three separate experiments.

The RNA ligase RtcB catalyzes the GTP and Mn(II)-dependent joining of 2′,3′-cyclic phosphate or 3′-phosphate termini to 5′-hydroxyl termini (4–12). RtcB is an essential enzyme for the ligation of tRNAs in metazoa (4), and likely archaea (5,13), after intron removal by the tRNA splicing endonuclease (14,15). Ligation proceeds through three nucleotidyl transfer steps, with 2′,3′-cyclic phosphate termini being hydrolyzed to 3′-P termini in a step that precedes 3′-P activation with GMP (7,9,10) (Figure 1A). In the first nucleotidyl transfer step, RtcB reacts with GTP to form a covalent RtcB–histidine–GMP intermediate and release PP_i_; in the second step, the GMP moiety is transferred to the RNA 3′-P; in the third step, the 5′-OH from the opposite RNA strand attacks the activated 3′-P to form a 3′,5′-phosphodiester bond and release GMP.

We sought to discover whether Archease and RtcB encoded within the archaeon *Pyrococcus horikoshii* could function in tandem. We envisioned that the hyperthermophilic archaeal proteins would be an excellent model system, due to their ease of purification and their inherent conformational stability. Moreover, the high conservation of Archease and RtcB across the three domains of life suggests that studies on the archaeal proteins would be broadly applicable (1,5). Here, we show that Archease and RtcB from *P. horikoshii* do indeed function in unison, and that Archease affects the catalytic properties of RtcB. Archease expands the cofactor specificity of RtcB, enabling the efficient use of dGTP, ATP or ITP. We also demonstrate that Archease accelerates the RNA 3′-P activation and ligation steps of catalysis by RtcB. In addition, our structural and mutational analyses of Archease identify an essential metal-binding site consisting of conserved carboxylate groups located at a tip of the protein. Together, these data demonstrate the existence of a tRNA splicing operon.

## MATERIALS AND METHODS

### Archease production and purification

The gene encoding *P. horikoshii* Archease (Accession #O59205) was synthesized by Integrated DNA Technologies using codons optimized for expression in *Escherichia coli*. The gene was cloned between the *Sph*I and *Bam*HI recognition sites of vector pQE70-*lacI* (Qiagen). Cloning into the *Sph*I site required insertion of a codon that begins with a cytosine immediately after the ATG start codon. A leucine codon was therefore inserted after ATG; however, throughout the manuscript we maintain the amino-acid numbering corresponding to native *P. horikoshii* Archease. The resulting plasmid was transformed into the BL21 strain of *E. coli*, and the Archease protein was produced by growing cells in Terrific Broth medium to an OD_600_ of 0.7, inducing gene expression by adding IPTG to 0.5 mM, and continuing growth for 3 h at 37°C. Cells were harvested by centrifugation and resuspended in 8 ml per gram of wet pellet in buffer A (50 mM Tris–HCl buffer, pH 8.6, containing 50 mM NaCl). Cells were lysed by passage through a cell disruptor (Constant Systems) at 20 000 psi, and the lysate was clarified by centrifugation at 20 000*g* for 1 h. Bacterial proteins were precipitated and removed by incubating the lysate at 70°C for 25 min, followed by centrifugation at 20 000*g* for 20 min. The clarified lysate was then loaded onto a 5-ml HiTrap Q XL anion-exchange column (GE Healthcare). The column was washed with 50 ml of buffer A, and Archease was eluted with a NaCl gradient of buffer A (50 mM–1.0 M) over 20 column volumes. Fractions containing purified Archease were dialyzed against 2 l of buffer (10 mM HEPES–NaOH, pH 7.5, containing 200 mM NaCl) overnight at 4°C, and the protein was flash-frozen in liquid nitrogen and stored at −80°C. Concentrations of RtcB and Archease were calculated from *A*_280_ values and calculated (ExPASy) extinction coefficients of *ε*_280_ = 62 340 M^−^^1 ^cm^−^^1^ and 19 940 M^−^^1 ^cm^−^^1^, respectively.

### RNA ligation and guanylylation assays

Native *P. horikoshii* RtcB was produced and purified as described previously (12). Ligation reaction mixtures included a 10-nt 5′ RNA fragment and a 10-nt 3′ RNA fragment. The 5′ RNA fragment had a 6-carboxyfluorescein (FAM) label on its 5′ end and was phosphorylated on its 3′ end. The 3′ RNA fragment had hydroxyl groups on each end. The sequence of the 5′ fragment was FAM-5′-AAAUAACAAA-3′-P, and the sequence of the 3′ RNA fragment was 5′-AAAUAACAAA-3′. Ligation reactions were performed at 70°C in 25 or 50 µl solutions consisting of 50 mM Bis–Tris buffer (pH 7.0), containing NaCl (300 mM), MnCl_2_ (0.25 mM), NTP (0.10 mM), *P. horikoshii* RtcB (5 μM), 5′ RNA fragment (1.0 μM) and 3′ RNA fragment (1.0 μM). RNA 3′-P guanylylation reactions were performed in identical reaction mixtures except the RNA ligation substrates were replaced with a 5′ RNA fragment (1.0 μM) that had a 2′-F replacing the 2′-OH that is vicinal to the terminal 3′-P. Reactions were quenched by the addition of an equal volume of RNA gel-loading buffer (5× TBE containing 7 M urea, 20% v/v glycerol and 15 mg/ml blue dextran). Reaction products were separated on an 18% w/v urea–polyacrylamide gel, and the RNA was visualized by fluorescence scanning with a Typhoon FLA9000 imager (GE Healthcare). Product quantification was performed using ImageQuant TL (GE Healthcare).

### [^14^C]GTP binding assays

Binding assays were performed in 250 μl solutions consisting of 50 mM Bis–Tris buffer (pH 7.0), containing NaCl (300 mM), MnCl_2_ (2.0 mM) and [8-^14^C]GTP (1.0 mM) (Moravek Biochemicals). RtcB was assayed at a concentration of 30 µM, and Archease was assayed at 100 µM. After incubation at 70°C, free GTP was removed by applying the reaction mixture to three 5-ml HiTrap desalting columns (GE Healthcare) connected in series. The desalting columns were equilibrated with elution buffer (50 mM HEPES–NaOH buffer, pH 7.5, containing 200 mM NaCl), and protein was eluted in 0.5-ml fractions. Absorbance readings at 260 and 280 nm were obtained for each fraction. The protein fractions have high *A*_280_ readings, whereas the fractions with free GTP have high *A*_260_ readings. The concentration of [8-^14^C]GTP in the protein fractions was determined by liquid scintillation counting. Each 0.5-ml fraction was mixed with 3.5 ml of Ultima Gold MV liquid scintillation cocktail (Perkin Elmer) in a 4-ml vial, and radioactivity was quantified with a MicroBeta TriLux liquid scintillation counter (Perkin Elmer). The concentration of GTP in each fraction was determined by comparing the counts per minute in these samples to that in standards of known concentration. Although we are assaying for GTP binding to RtcB, we are assuming that the radioactivity remaining bound to RtcB is bound covalently in the form of RtcB–pG, as has been shown previously (11,12).

### Archease crystallization, data collection and structure determination

Selenomethionine labeled (Se-Met) Archease was produced in the *E. coli* methionine auxotroph strain B834 in auto-induction medium (16). Archease was purified as described above, concentrated to 2.9 mM (48 mg/ml) by ultrafiltration using a spin concentrator (5000 MWCO, Amicon). The protein was flash-frozen in liquid nitrogen and stored at −80°C. Archease crystals diffracted to a higher resolution when MgCl_2_ (11.5 mM) was added to the protein solution before crystallization. Archease was crystallized using the hanging drop vapor diffusion method. Crystals were grown by mixing 1 μl of protein solution with 1 μl of reservoir solution consisting of sodium acetate (0.10 M, pH 4.5), (+/−)-2-methyl-2,4-pentanediol (40% v/v) and CaCl_2_ (10 mM). Trays were incubated at 20°C and crystals appeared within 1 week. Crystals were harvested, cryoprotected in MiTeGen low viscosity cryo oil and flash-frozen in liquid nitrogen. X-ray diffraction data were collected at the General Medicine and Cancer Institutes Collaborative Access Team (GM/CA-CAT) at Argonne National Laboratory. Data sets were collected at the selenium peak and edge wavelengths from a single crystal. Data sets were indexed and scaled using HKL2000 (17). The structure was solved using Phenix Autosol (18) and completed using alternating rounds of manual model building using COOT (19) and refinement with phenix.refine (18). Structure quality was assessed with MolProbity (20), and figures were generated using PyMOL (21). Omit maps were calculated with Phenix.

### Sedimentation equilibrium analysis

Sedimentation equilibrium studies were performed with a Beckman Optima XL-A analytical ultracentrifuge in the Biophysics Instrumentation Facility at the University of Wisconsin–Madison. The identical Se-Met Archease preparation used for crystallography was analyzed at concentrations of 8.4, 16.7 and 25 μM in HEPES–NaOH (10 mM, pH 7.5), containing NaCl (200 mM). A run that included MnCl_2_ (0.25 mM) was also performed. Equilibrium data were collected at multiple speeds at 20°C.

## RESULTS

### Archease activates RtcB

Genomic context analysis shows that the *archease* gene is most commonly located directly adjacent to the *rtcB* gene and the two genes are localized into an operon in diverse bacteria and archaea (22,23) (Figure 1B). Moreover, the gene encoding a tRNA m^5^C methyltransferase is sometimes found in the same operon as *rtcB* and *archease*, providing an additional clue that *archease* is involved in tRNA maturation (2). To investigate a possible functional interaction between Archease and RtcB from *P. horikoshii*, the *archease* gene was synthesized using codons optimized for expression in *E. coli*. Soluble Archease was produced at high levels—we isolated ∼80 mg of protein routinely from a 1 -l culture (see ‘Materials and Methods’ section). To assay for RtcB-catalyzed RNA ligation, we used two 10-nt RNA substrates that can be joined to produce a 20-nt product. The 5′ RNA half contains a 5′ fluorescent label and a 3′-P, whereas the 3′ RNA half has hydroxyl groups at each terminus (Figure 1C). When Archease was titrated into RNA ligation reaction mixtures containing 5 μM RtcB and 1.0 μM of each RNA half, the extent of RtcB activation leveled off at an Archease concentration of 100 nM (Figure 1C and D). An Archease concentration of 100 nM enabled 5 μM RtcB to produce 316 nM of ligation product, whereas only 92 nM of product was formed in the absence of Archease (Figure 1D). Thus, 100 nM Archease enabled 5 μM RtcB to produce 224 nM *more* ligation product. The ability of a sub-stoichiometric ratio of Archease:RtcB to enable the formation of additional ligation product that exceeds the concentration of Archease, suggests that Archease acts catalytically. Reactions to determine the pH-dependence of RtcB activation by Archease showed that maximal activation occurs at pH 7.0 (Supplementary Figure S1). Control reactions that did not include RtcB demonstrated that Archease does not have RNA 3′-P guanylylation or ligation activity (data not shown).

### Effect of Archease on RNA ligation kinetics

**Figure 2.**
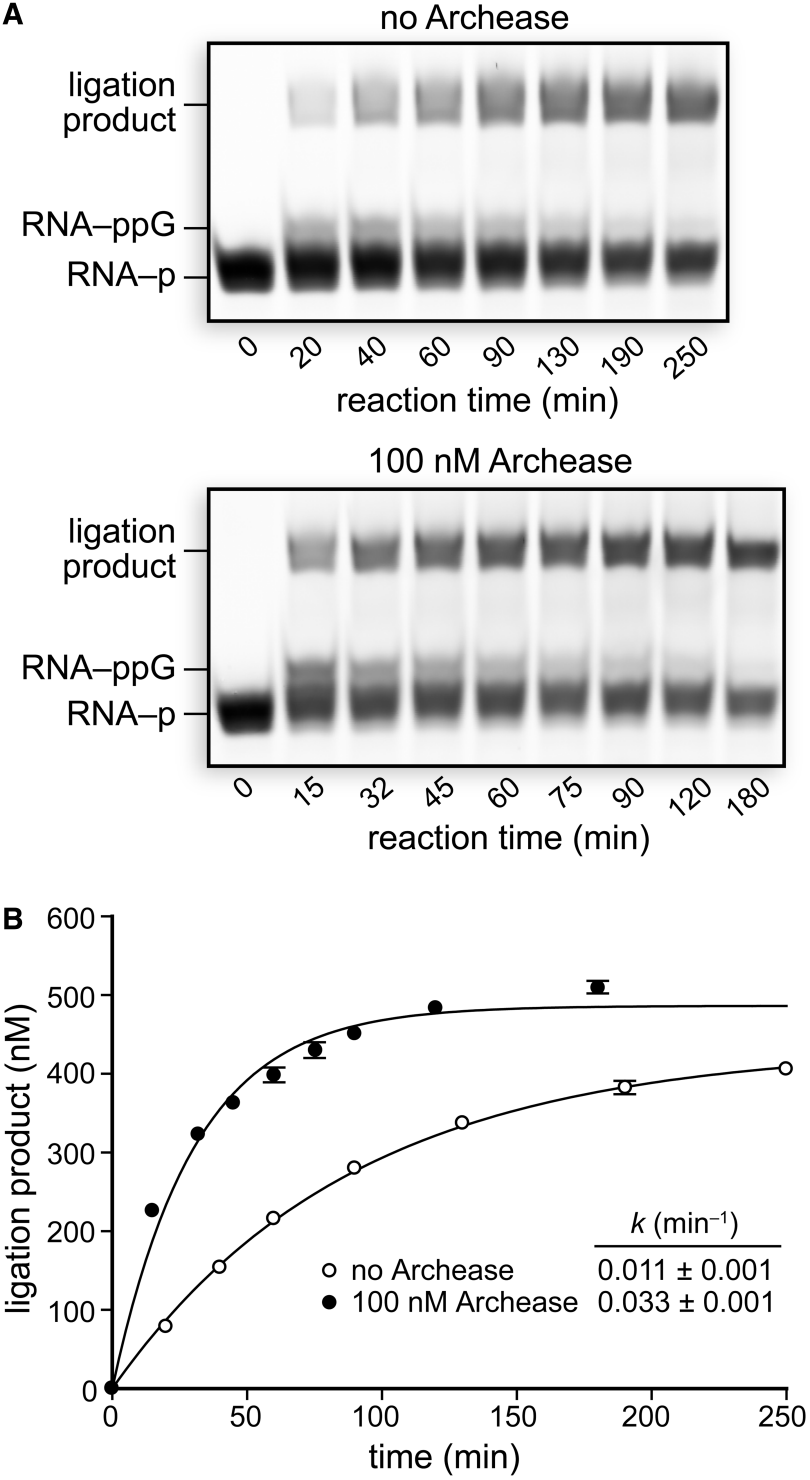
Effect of Archease on the single-turnover rate of RNA ligation by RtcB. (**A**) RNA ligation reactions with RtcB alone or with the inclusion of 100 nM Archease. Reaction mixtures contained 50 mM Bis–Tris buffer (pH 7.0), NaCl (300 mM), MnCl_2_ (0.25 mM), GTP (0.10 mM), *P. horikoshii* RtcB (5 μM), 5′ RNA fragment (1.0 μM) and 3′ RNA fragment (1.0 μM). Reaction mixtures were incubated at 70°C, and aliquots were removed at the indicated times and quenched with an equal volume of RNA gel-loading buffer. (**B**) Plots of ligation product formation over time fitted to a single-exponential equation. Values are the mean ± SE for two separate experiments.

Next, we examined the effect of Archease on the overall rate of the three-step RtcB ligation pathway by monitoring the rate of ligation over time under single-turnover conditions. Reaction mixtures containing 5 μM RtcB alone and including 100 nM Archease were incubated at 70°C, and aliquots were removed and quenched at various time intervals (Figure 2A). Plots of the concentration of ligation product formed over time were fitted to a single-exponential to obtain apparent rate constants of (0.011 ± 0.001) min^−^^1^ for RtcB alone and (0.033 ± 0.001) min^−^^1^ with the inclusion of Archease (Figure 2B). Thus, 100 nM Archease accelerates the overall three-step RtcB ligation pathway by 3-fold under our reaction conditions. Despite having RtcB in 5-fold excess over RNA substrates, the ligation reaction with RtcB alone went to only 43% completion and the reaction with Archease included went to only 49% completion (Figure 2B). The ability for only ∼10% of RtcB molecules to catalyze a ligation event suggests that RtcB has a preferred RNA substrate or that the system requires unknown components for increased ligation efficiency.

### Effect of Archease on RNA 3′-P guanylylation kinetics

Then, we analyzed the effect of Archease on the rate of RNA 3′-P guanylylation. To monitor RNA 3′-P guanylylation directly, we envisioned that we could first react RtcB with GTP and Mn(II) to form the RtcB–pG intermediate and also exclude an RNA 5′-OH terminus from the reaction, thus eliminating the first and third nucleotidyl transfer steps, respectively. We first demonstrated that Archease could activate RNA ligation when RtcB is replaced with RtcB–pG. Using [^14^C]GTP, we found that RtcB is converted fully to RtcB–pG within 5 min when incubated with GTP (1.0 mM) and MnCl_2_ (2.0 mM) at 70°C (see ‘Materials and Methods’ section). The RtcB–pG intermediate was then subjected to the action of Archease in RNA ligation reactions. We observed that substituting RtcB with the preformed RtcB–pG intermediate had little effect on Archease-dependent activation (Supplementary Figure S2).

**Figure 3.**
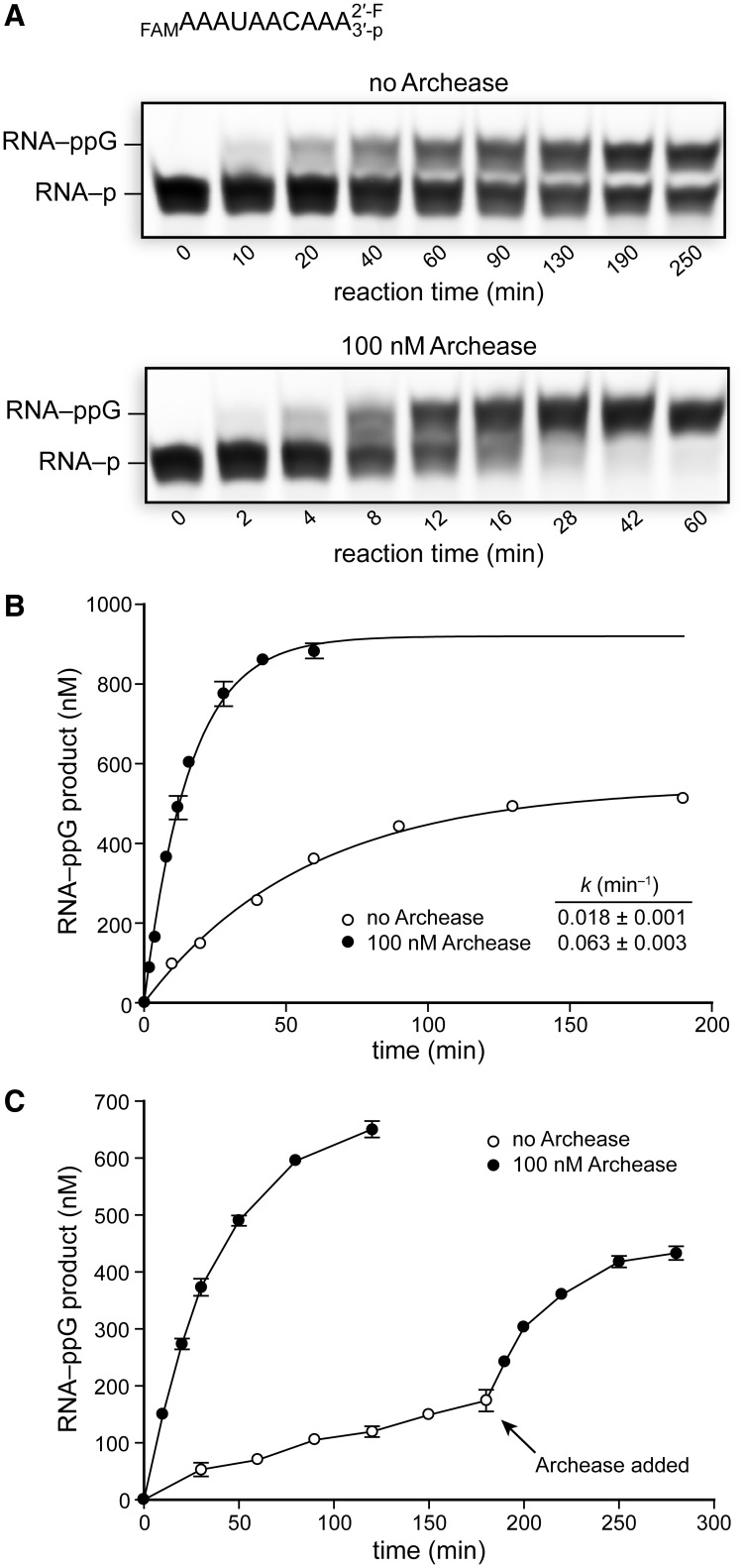
Effects of Archease on the rate of RtcB-catalyzed guanylylation of RNA with a 2′-F/3′-P terminus. RtcB was pre-guanylylated by incubation with GTP and Mn(II), and the 3′ RNA fragment was not included to prevent ligation. (**A**) The guanylylation rate of a 2′-F/3′-P RNA terminus by RtcB alone or with the inclusion of 100 nM Archease. RNA guanylylation reaction mixtures contained 50 mM Bis–Tris buffer (pH 7.0), NaCl (300 mM), MnCl_2_ (0.25 mM), GTP (0.10 mM), *P. horikoshii* RtcB (5 μM) and RNA substrate (1.0 μM). (RNA substrate is shown at top.) Reaction mixtures were incubated at 70°C, and aliquots were removed at the indicated times and quenched with an equal volume of RNA gel-loading buffer. (**B**) Plots of RNA–ppG product formation over time fitted to a single-exponential equation. (**C**) Plots of RtcB-catalyzed RNA–ppG product formation over time in reactions that had the 2′-F/3′-P RNA substrate in excess (0.5 μM RtcB and 1.0 μM RNA substrate). Archease (100 nM) was added where indicated. Values in the plots are the mean ± SE for two separate experiments.

The activated RNA–ppG intermediate could be captured stably if RNA with a 2′-F/3′-P terminus was used as the guanylylation substrate, thus facilitating kinetic analyses. RNA 3′-P guanylylation reaction mixtures containing 1.0 μM RNA and 5 μM RtcB alone and including 100 nM Archease were incubated at 70°C, and aliquots were removed and quenched at various time intervals (Figure 3A). The concentration of RNA–ppG product formed over time was plotted and fitted to a single-exponential to obtain apparent rate constants of (0.018 ± 0.001) min^−^^1^ for RtcB alone and (0.063 ± 0.003) min^−^^1^ in the presence of 100 nM Archease (Figure 3B). Thus, Archease at 100 nM accelerates the RNA 3′-P guanylylation step by 3.5-fold under our reaction conditions. Significantly, Archease enabled the RNA 3′-P guanylylation reaction to reach near completion (>90%), whereas the reaction in the absence of Archease went to only 54% completion. In the absence of Archease, only 10% of RtcB molecules are competent for catalyzing RNA 3′-P guanylylation, a finding similar to our observed ligation efficiency. The rate constants obtained for RNA 3′-P guanylylation are faster than those obtained for the overall ligation pathway, consistent with RNA–ppG being a kinetically competent intermediate. Considering that formation of the RtcB–pG intermediate is fast, comparison of the apparent rate constants demonstrates that the ligation step is rate-limiting under the tested reaction conditions. Thus, Archease accelerates both the second and the third nucleotidyl transfer steps of catalysis by RtcB.

The apparent stalling of the RNA 3′-P guanylylation reaction in the absence of Archease suggests that not all the RtcB molecules are active for the second nucleotidyl transfer step or that RtcB arrests after the histidine guanylylation step. The increase in reaction completion when Archease is included could indicate that Archease functions by activating stalled RtcB molecules. The effect of Archease on reaction completion should become more apparent when the concentration of RtcB is less than the concentration of substrate RNA. In reactions with 0.5 μM RtcB and 1.0 μM RNA 3′-P the reaction rate was sluggish and only 30% of RtcB molecules catalyzed a single turnover after 3 h at 70°C (Figure 3C). When Archease was included, 0.5 μM RtcB produced 0.65 μM RNA–ppG within 2 h, consistent with 100% of enzyme molecules catalyzing at least one turnover. Moreover, the reaction in the absence of Archease was activated rapidly on Archease addition (Figure 3C). This experiment demonstrated that each RtcB molecule is competent for catalyzing RNA 3′-P guanylylation when Archease is included.

The formation of activated RNA 3′-P at a concentration that exceeds the RtcB concentration suggests that the ligase can release the activated RNA into solution. The RNA–ppG released into solution would be expected to form a 2′,3′-cyclic phosphate (RNA > p) rapidly via intramolecular attack by the terminal 2′-OH, releasing GMP. The RNA > p could again be a competent substrate for RtcB–pG. In contrast, the release of activated RNA or DNA by classical ATP-dependent ligases poses a great challenge to cells (24,25). The released adenylylated RNA or DNA intermediate is not a ligase substrate because the ligase reacts quickly with ATP to form ligase–pA, thus occupying the AMP-binding pocket. Hence, essential repair pathways are needed to remove the nucleic acid 5′-adenylyl group so as to regenerate a competent ligase substrate (25).

### Archease expands the NTP cofactor specificity of RtcB

**Figure 4.**
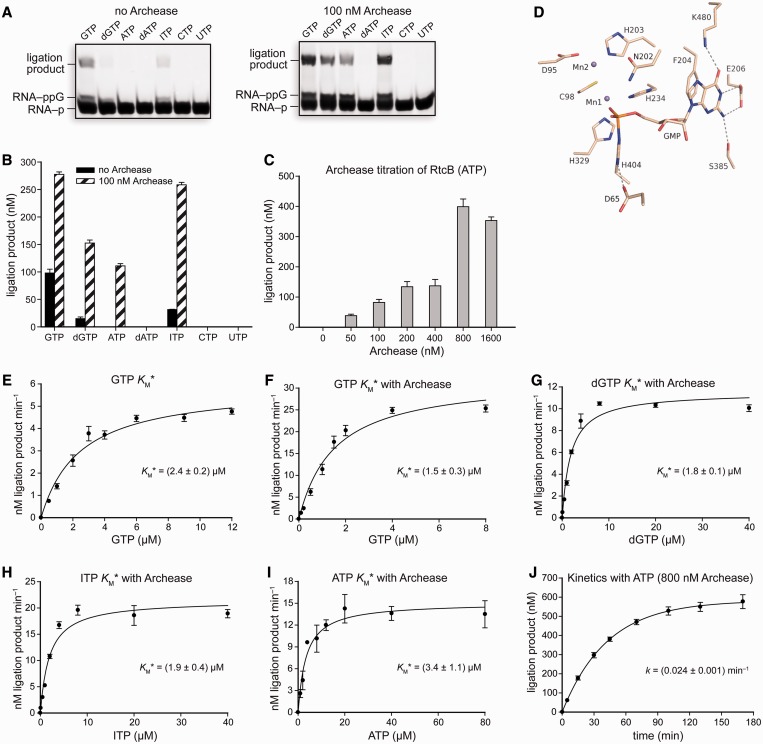
RNA ligation reactions demonstrating the NTP cofactor specificity of RtcB and an active-site view of the RtcB–pG intermediate. (**A**) Reactions testing NTP cofactor specificity with RtcB alone or RtcB with 100 nM Archease. NTP cofactors were tested at 0.10 mM, and reaction mixtures were incubated at 70°C for 30 min. (**B**) Graph of the ligation product obtained for each NTP cofactor. Values are the mean ± SE for two separate experiments. (**C**) ATP-dependent RtcB-catalyzed RNA ligation reactions titrated with increasing concentrations of Archease, as specified. Reaction mixtures were incubated at 70°C for 20 min. Values are the mean ± SE for three separate experiments. (**D**) Crystal structure of the *P. horikoshii* RtcB–pG intermediate (PDB entry 4it0) illustrating the residues that contact the guanine nucleobase. (**E–I**) Michaelis–Menten plots of reaction rate versus NTP cofactor concentration for RtcB-catalyzed RNA ligation reactions under single-turnover conditions. Where indicated, Archease was included at a concentration of 100 nM for reactions with GTP, dGTP and ITP, while reactions with ATP included 800 nM Archease. Values are the mean ± SE for three separate experiments. (**J**) Single-turnover kinetics of ATP-dependent RNA ligation catalyzed by RtcB with the inclusion of Archease (800 nM). Values are the mean ± SE for two separate experiments. Ligation reaction mixtures contained 50 mM Bis–Tris buffer (pH 7.0), NaCl (300 mM), MnCl_2_ (0.25 mM), NTP as indicated, *P. horikoshii* RtcB (5 μM), 5′ RNA fragment (1.0 μM) and 3′ RNA fragment (1.0 μM).

While investigating a possible Archease-dependent effect on the first nucleotidyl transfer step of catalysis by RtcB, we first discovered that Archease enhances the weak dGTP-dependent ligation activity of RtcB. Then, we wondered whether Archease could also enable RtcB to use NTPs with different nucleobases. We tested ATP, dATP, ITP, CTP and UTP at 0.10 mM in ligation reactions with RtcB. In the absence of Archease, RtcB-catalyzed RNA ligation proceeds efficiently with GTP and substantially less efficiently with dGTP and ITP (Figure 4A and B). Notably, inclusion of Archease in ligation reaction mixtures enabled efficient utilization of GTP, dGTP, ATP or ITP (Figure 4A and B). Thus, Archease enabled RtcB to use all tested purine nucleobases, though not the pyrimidines CTP and UTP. We also observed that the amount of ligated product obtained with ATP was greatest when the Archease concentration was increased to 800 nM (Figure 4C). Archease increased the amount of ligation product formed with the cofactors dGTP and ITP by 10 - and 8-fold, respectively, during incubation of the reaction mixtures at 70°C for 30 min. The ability for RtcB ligation to proceed with ATP is confounding, given the hydrogen-bonding constraints to the nucleobase apparent in the crystal structure of the RtcB–pG intermediate (11,12) (Figure 4D). Importantly, we did not observe binding of [^14^C]GTP to Archease, suggesting that it is unable to bind and deliver purine NTPs directly to the RtcB active site (see ‘Materials and Methods’ section).

The NTP dependence of RtcB and RtcB·Archease under single-turnover conditions appeared to follow Michaelis–Menten behavior, allowing determination of NTP cofactor Michaelis constant values (*K*_M_* is defined as the apparent *K*_M_ value under single-turnover reaction conditions, Figure 4E–I). The GTP *K*_M_* values for RtcB and RtcB·Archease are (2.4 ± 0.2) and (1.5 ± 0.3) μM, respectively. The dGTP and ITP *K*_M_* values for RtcB·Archease are (1.8 ± 0.1) and (1.9 ± 0.4) μM, respectively. We were unable to determine *K*_M_* values for dGTP and ITP in the absence of Archease owing to their low reactivity. Nevertheless, the ATP *K*_M_* value for RtcB·Archease is (3.4 ± 1.1) μM, demonstrating efficient use of this cofactor. The ATP *K*_M_ values for human DNA ligase 1 and T4 DNA ligase are 12 and 14 μM, respectively (24,26). The apparent rate constant for the overall ligation pathway with ATP including 800 nM Archease is (0.024 ± 0.001) min^−^^1^ (Figure 4J).

### Crystal structure of *P. horikoshii* Archease

**Figure 5.**
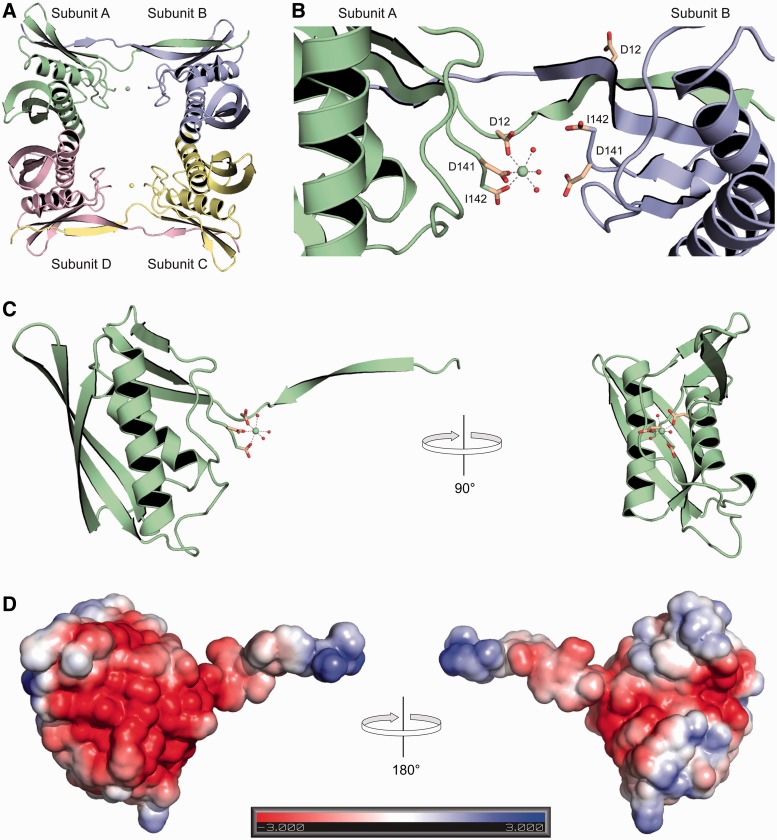
Crystal structure of *P. horikoshii* Archease. (**A**) A cartoon representation of the four Archease subunits per asymmetric unit with the Ca(II) ions represented as spheres. (**B**) The Ca(II) ion-binding site at the interface of subunits A and B. The Ca(II) ion-binding residues are depicted as sticks, and the Ca(II) ion and water molecules are shown as spheres. (**C**) Archease subunit A depicting the Ca(II) ion-binding site at the base of the N-terminal protrusion. A turn of 90° demonstrates the slenderness of the protein. (**D**) Electrostatic surface potential of Archease subunit A with blue and red indicating regions of positive and negative charge, respectively.

To gain greater insight into the mechanism of Archease action, we solved an x-ray crystal structure of *P. horikoshii* Archease. Archease crystals diffracted to a resolution of 1.4 Å and contained four protein molecules per asymmetric unit (Supplementary Table S1). Archease appeared to crystallize as a dimer of dimers; however, sedimentation equilibrium results were consistent with the existence of a predominantly monomeric species in both the presence and absence of Mn(II) (Figure 5A). Archease subunits A and B are essentially identical to subunits C and D, respectively. The structure of each subunit consists of two strand–helix–strand domains, each consisting of a three-strand core and a single helix in a strand–helix–strand configuration (27). The crystallization buffer included CaCl_2_ and the omit density map showed two Ca(II) ions per asymmetric unit, with each Ca(II) ion bound to a single Archease subunit in octahedral coordination geometry (Supplementary Figure S3). The metal-binding sites are identical and consist of two strictly conserved aspartate residues, the C-terminal carboxylate group and three water molecules (Figure 5B and Supplementary Figure S4). The metal-binding site is located at the interface between two subunits, subunit A binds a Ca(II) ion while the analogous residues in subunit B are not in a position for metal binding (Figure 5B). The N and C termini of each Archease subunit are proximal, and the N terminus exists as an extended protrusion that forms a beta sheet with its partner subunit. The Ca(II)-binding site resides at the base of the N-terminal protrusion on the protein exterior. Rotation of the Archease structure demonstrates that the small protein is slender, spanning only ∼20 Å on one side (Figure 5C). The electrostatic surface potential of Archease is dominated by regions of negative charge (Figure 5D).

### Structure-guided mutagenesis of Archease

**Figure 6.**
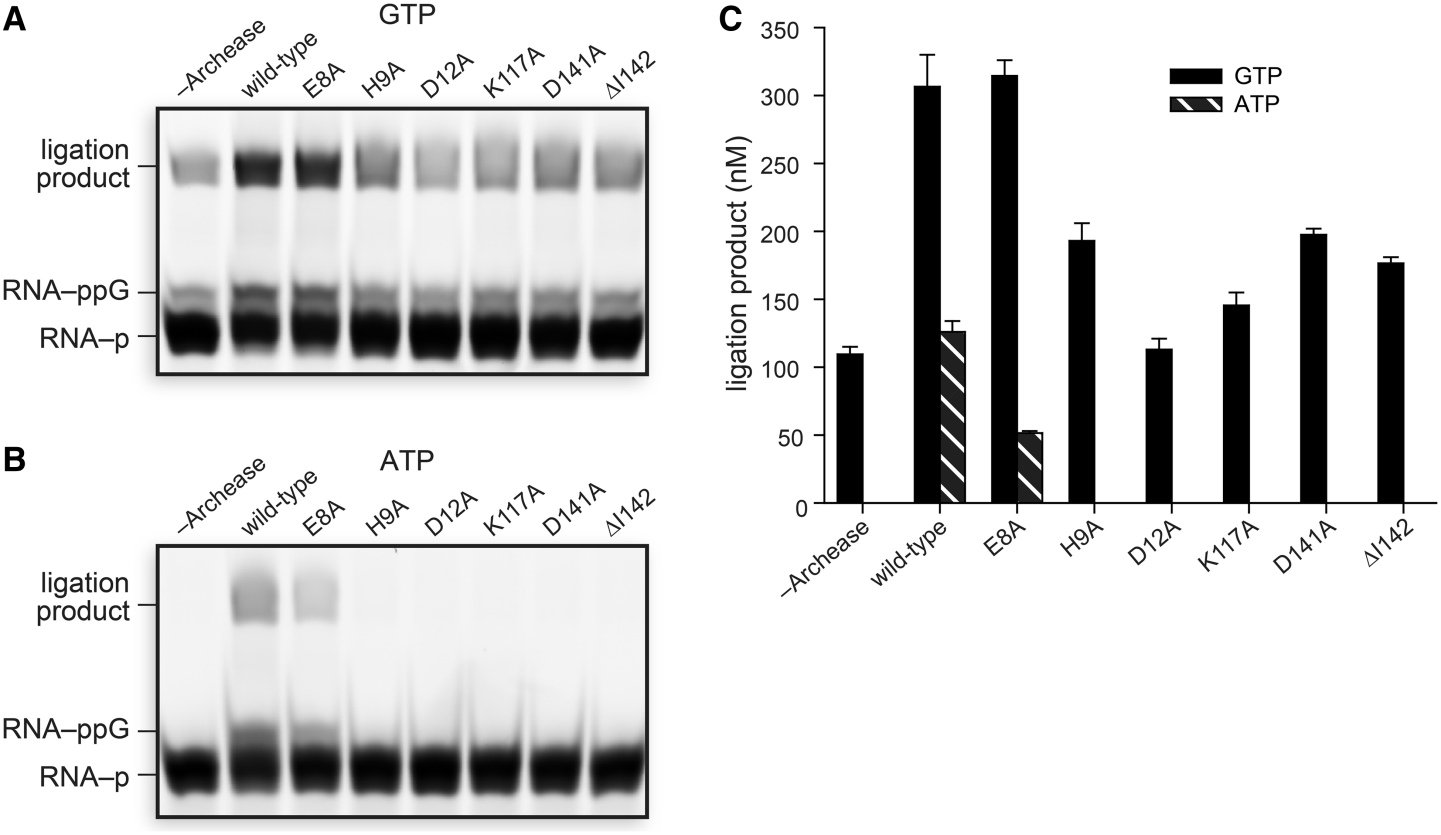
Structure-guided mutagenesis of conserved Archease residues. (**A**, **B**) Archease variants with alanine substitutions were tested for their ability to activate RtcB in RNA ligation reactions with GTP (A) or ATP (B) as a cofactor. Reaction mixtures included 100 nM Archease where specified and were incubated at 70°C for 30 min. (**C**) Graph of the ligation product obtained for each Archease variant. Values are the mean ± SE for two separate experiments. Ligation reaction mixtures contained 50 mM Bis–Tris buffer (pH 7.0), NaCl (300 mM), MnCl_2_ (0.25 mM), GTP or ATP (0.10 mM), *P. horikoshii* RtcB (5 μM), 5′ RNA fragment (1.0 μM) and 3′ RNA fragment (1.0 μM).

Site-directed mutagenesis of residues in the *P. horikoshii* Archease metal-binding site revealed their importance for the Archease-dependent activation of RtcB. The metal-binding variants D12A, D141A and ΔI142, drastically reduced the Archease-dependent activation of RtcB (Figure 6A and Supplementary Figure S5). The D12A Archease variant had the most diminished RtcB-activation activity. Residue His9 is a highly conserved residue in the N-terminal tail, and Lys117 is a highly conserved residue adjacent to the metal-binding site. Substitution of each of these two residues also severely reduced Archease-dependent activation of RtcB, though an E8A substitution had no detrimental effect. The effects of the Archease substitutions in the metal-binding site were recapitulated when ATP was used as a cofactor (Figure 6B and C). In contrast to the observation with GTP, the E8A variant of Archease had a substantial effect on ATP utilization by RtcB.

### Archease rescues inactive RtcB variants that have substitutions in the guanine-binding pocket

**Figure 7.**
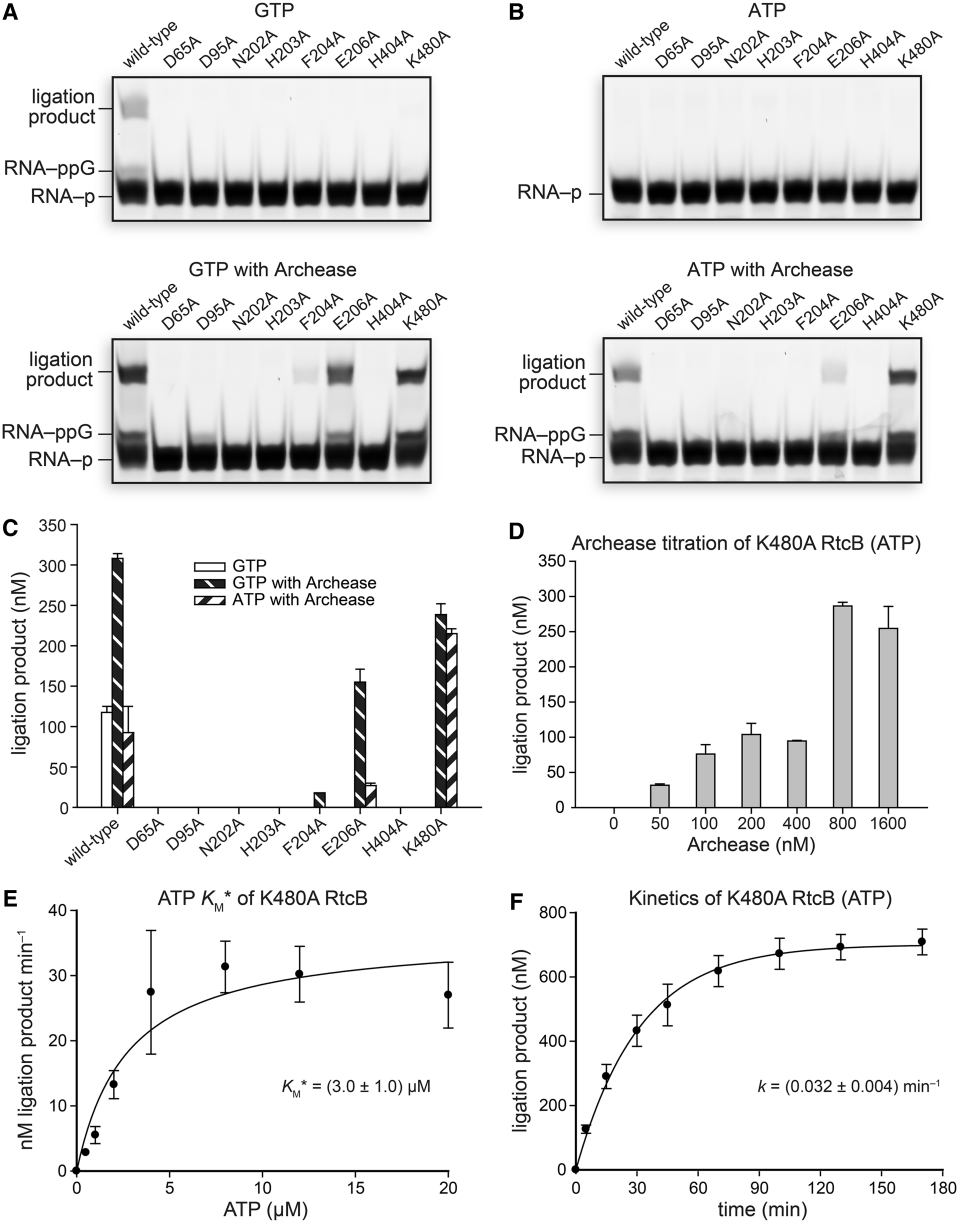
Effect of Archease on the activity of active-site variants of RtcB in RNA ligation assays with GTP or ATP as a cofactor. (**A**) Reactions with GTP (0.10 mM) as a cofactor. (**B**) Reactions with ATP (0.10 mM) as a cofactor. Reaction mixtures included 100 nM Archease where indicated, and were incubated at 70°C for 30 min. (**C**) Graph of the ligation product obtained for each RtcB variant. Values are the mean ± SE for two separate experiments. (**D**) ATP-dependent K480A RtcB-catalyzed RNA ligation reactions titrated with increasing concentrations of Archease, as specified. ATP was included at 0.10 mM, and reaction mixtures were incubated at 70°C for 15 min. Values are the mean ± SE for three separate experiments. (**E**) Michaelis–Menten plot of reaction rate versus ATP cofactor concentration for K480A RtcB-catalyzed RNA ligation reactions under single-turnover conditions. Values are the mean ± SE for three separate experiments. (**F**) Single-turnover kinetics of ATP-dependent RNA ligation catalyzed by K480A RtcB with the inclusion of Archease (800 nM). Values are the mean ± SE for two separate experiments. Ligation reaction mixtures contained 50 mM Bis–Tris buffer (pH 7.0), NaCl (300 mM), MnCl_2_ (0.25 mM), NTP as indicated, *P. horikoshii* RtcB (5 μM), 5′ RNA fragment (1.0 μM) and 3′ RNA fragment (1.0 μM).

If Archease is perturbing the binding of the NTP nucleobase, we reasoned that residues interacting with guanine, as observed in the RtcB–pG crystal structure, would become irrelevant for catalysis by RtcB in the presence of Archease. To test this hypothesis, active-site variants of RtcB were assayed in ligation reactions with and without the inclusion of Archease. The RtcB amino-acid substitutions assayed were D65A, D95A, N202A, H203A, F204A, E206A, H404A and K480A. Each of these substitutions rendered RtcB alone inactive. Yet, when Archease was included, the F204A, E206A and K480A variants were rescued—they became active catalysts of the ligation reaction (Figure 7A). These three variants had substitutions of residues that interact directly with the guanine base in the RtcB–pG structure (11,12) (Figure 4D). The ligation activity of RtcB variants with substitutions of residues involved directly in binding Mn(II) (D95A and H203A), interacting with the triphosphate moiety (N202A), and forming the histidine–GMP covalent bond (H404A and D65A) were unable to be rescued by Archease. We did, however, observe that Archease rescued the RNA 3′-P guanylylation activity of D95A RtcB (Figure 7A), a variant perturbed in the binding site for the second Mn(II) ion, Mn2 (Figure 4D). The rescue of ligation activity with GTP as a cofactor was recapitulated with ATP as a cofactor, except for undetectable activity with F204A RtcB (Figure 7B and C). When assayed with ATP as a cofactor, K480A RtcB catalyzed the formation of ∼2-fold more ligation product than did the wild-type enzyme in the presence of Archease during incubation of the reaction mixtures at 70°C for 30 min. This finding suggested that eliminating the clash between the amino group of Lys480 and the adenine exocyclic amine facilitates ATP utilization by lowering its *K*_M_ value. Yet, the ATP *K*_M_* value for K480A RtcB was nearly identical to the ATP *K*_M_* value for wild-type RtcB when 800 nM Archease was included (Figure 7D and E). Instead, the increased ligation product formed by K480A RtcB can be explained by an increased reaction rate under single-turnover RNA ligation conditions (Figure 7F). The apparent overall ligation rate constant for K480A RtcB with ATP was (0.032 ± 0.004) min^−^^1^, a value 33% greater than that obtained for wild-type RtcB with ATP under identical reaction conditions (Figure 4J).

## DISCUSSION

We have demonstrated the evolutionary rationale for organizing *rtcB* and *archease* into an operon in diverse bacteria and archaea. Likewise, the *E. coli* operon organization of *rtcA* (RNA 3′-terminal phosphate cyclase A) and *rtcB* was initially used as a rationale to test RtcB for the ability to ligate RNA 2′,3′-cyclic phosphate and 5′-OH termini (28), an activity which was confirmed later (6). RtcB and Archease are highly conserved in all three domains of life, and RtcB is known to be the essential catalytic component of a tRNA splicing complex in humans (4). Our functional studies of Archease and RtcB from *P. horikoshii* have shown that Archease modulates all three nucleotidyl transfer steps during catalysis by RtcB. It is especially notable that Archease converts RtcB from a ligase that displays no ATP-dependent activity to a ligase displaying low micromolar *K*_M_* values for GTP, dGTP, ITP and ATP. This demonstrates that Archease affects binding not only to the nucleobase, but also to the ribose. The ability for Archease to endow RtcB with altered NTP specificity, as well as recover the activity of RtcB variants with substitutions in the guanine-binding pocket, suggests that there might be a purine-binding pocket on Archease. We were, however, unable to detect direct binding of [^14^C]GTP to Archease. Alternatively, a novel composite purine-binding pocket could form on interaction of the two proteins, or Archease could enforce an alternative nucleoside binding conformation within the RtcB active site. The small size and negative surface charge of Archease are consistent with its binding in the positively charged cleft of RtcB, which is also presumed to be the binding site for substrate RNA. Elucidation of the mechanism of altered NTP specificity will likely require obtaining a crystal structure of the RtcB–pA·Archease complex.

Although we have identified an essential metal-binding site in Archease, we cannot ascertain if that site binds to its own metal ion or to one of the two Mn(II) ions in the RtcB active site. We do note that a metal-binding site consisting of absolutely conserved carboxylates located at the tip of Archease is reminiscent of GreB, a bacterial RNA polymerase transcription factor (29). GreB is a small (18.5 kDa) protein that functions to rescue a stalled RNA polymerase complex. RNA polymerase arrests when the transcript 3′-end loses base-pair contact with the DNA template, thereby disengaging the 3′-OH from the active site. GreB stimulates endonucleolytic cleavage of the transcript 3′-end, allowing RNA polymerase to restart. GreB has two conserved carboxylates at the tip of a coiled-coil that are placed into the polymerase active site and stabilize binding of the polymerase Mg2 ion, which stimulates an intrinsic endonucleolytic transcript cleavage activity (29,30).

Previously, we showed that RtcB uses a two-metal mechanism during reaction with GTP, a mechanism that is similar among all nucleotidyl transferases, including RNA polymerase (12,31). By analogy to GreB, the ‘sticky finger’ carboxylates of Archease might function to position or reposition an Mn(II) ion in the RtcB active site. The structure we present of Archease appears to capture it in a position appropriate for ‘handing off’ its metal ion to a partner protein (Figure 5B). A role for Archease in positioning Mn(II) in the Mn2 site is suggested by the observation that Archease can rescue the RNA 3′-P guanylylation activity of an RtcB variant that has an inactive D95A substitution in that site (Figure 7A). During the RtcB histidine-guanylylation reaction, Mn(II) in the Mn2 site reduces the negative charge on the GTP triphosphate moiety and orients the PP_i_ leaving group apically to the histidine nucleophile. The subsequent RNA 3′-P guanylylation reaction would require the RNA 3′-P to bind where PP_i_ was located, directly adjacent to the Mn2 site, such that an in-line attack can occur on the phosphorous atom of GMP. It is unknown whether Mn(II) leaves the Mn2 site along with PP_i_ or stays bound to RtcB. If Mn(II) is indeed displaced after histidine guanylylation, then the remarkable rescue of RNA 3′-P guanylylation activity by Archease could be explained by an effect on the Mn2 site. Additionally, the finding that only catalytic concentrations of Archease are required for RtcB activation suggests that the two proteins interact only transiently, with RtcB recruiting Archease as necessary.

An alternative hypothesis is that Archease assists in the release of GMP or ligated RNA formed as a product of the RtcB ligation step (Figure 1A). Product release is thought to be the rate-limiting step for nick-sealing by T4 DNA ligase (32). Yet, the rate accelerations we observed were under single-turnover conditions—the reaction rates were not dependent on GMP or ligated RNA product release. Also pertinent to consider is that Archease could affect the release of PP_i_ generated on formation of RtcB–pG. Obtaining a crystal structure of the RtcB·Archease complex will likely be important for understanding the mechanism of RtcB activation.

Activators of enzymatic activity are typically small molecules (33). Posttranslational modifications such as phosphorylation and acetylation can also enhance enzymatic activity (34–36). Protein activators of enzymes are rare, though the activation of a nucleic acid ligase by a protein partner is known. The bacterial proteins polynucleotide kinase-phosphatase (Pnkp) and hua enhancer 1 (Hen1) form a complex that repairs ribotoxin-cleaved RNAs. The Pnkp/Hen1 complex has evolved such that the methyltransferase Hen1 is required for activating the ligase activity of Pnkp. This requirement ensures that Hen1 has the opportunity to methylate the 2′-OH at the repair junction, thus preventing future ribotoxin-cleavage at the same site (37). The activity of eukaryotic DNA ligase IV, involved in nonhomologous end-joining, is activated by the proteins XRCC4 and XLF (38,39). The previous demonstration that Archease also modulates the specificity of a tRNA m^5^C methyltransferase (2) strengthens our conclusion that Archease is a critical factor for tRNA maturation. Here, we have demonstrated that Archease not only activates a tRNA ligase, but also broadens its NTP specificity.

## ACCESSION NUMBERS

PDB 4n2p.

## SUPPLEMENTARY DATA

Supplementary Data are available at NAR Online.

## FUNDING

National Institutes of Health [F32GM100681 to K.K.D]; Protein Structure Initiative grants [U01GM098248 to G.N.P.] and [U54GM074901 Center for Eukaryotic Structural Genomics]; [R01CA073808 to R.T.R]; Federal funds from the National Cancer Institute and the National Institute of General Medical Sciences [Y1-CO-1020 and Y1-GM-1104, respectively, toward the General Medicine and Cancer Institute Collaborative Access Team (GM/CA-CAT)]; US Department of Energy, Basic Energy Sciences, Office of Science [DE-AC02-06CH11357 toward use of the Advanced Photon Source]. Funding for open access charge: NIH [R01CA073808].

*Conflict of interest statement*. None declared.

## Supplementary Material

Supplementary Data
