# Supplementary material for: A tRNA splicing operon: Archease endows RtcB with dual GTP/ATP cofactor specificity and accelerates RNA ligation
Source: Nucleic Acids Res. 2014 Jan 16;42(6):3931–42. doi: 10.1093/nar/gkt1375 (PMC3973293; doi:10.1093/nar/gkt1375)
Supplement: Supplementary Data [file supp_42_6_3931__index.html]

A tRNA splicing operon: Archease endows RtcB with dual GTP/ATP cofactor specificity and accelerates RNA ligation — A tRNA splicing operon: Archease endows RtcB with dual GTP/ATP cofactor specificity and accelerates RNA ligation — Supplementary Data 

# A tRNA splicing operon: Archease endows RtcB with dual GTP/ATP cofactor specificity and accelerates RNA ligation

## Supplementary Data

files

**Files in this Data Supplement:**

- Supplementary Data - pdf file
