# Supplementary material for: A tRNA splicing operon: Archease endows RtcB with dual GTP/ATP cofactor specificity and accelerates RNA ligation
Source: Nucleic Acids Res. 2014 Jan 16;42(6):3931–42. doi: 10.1093/nar/gkt1375 (PMC3973293; doi:10.1093/nar/gkt1375)
Supplement: Supplementary Data [file supp_gkt1375_nar-03199-v-2013-File009.pdf]

A tRNA splicing operon: Archease endows RtcB with dual GTP/ATP cofactor specificity and accelerates RNA ligation

Kevin K. Desai, Chin L. Cheng, Craig A. Bingman, George N. Phillips, Jr.,

and Ronald T. Raines*

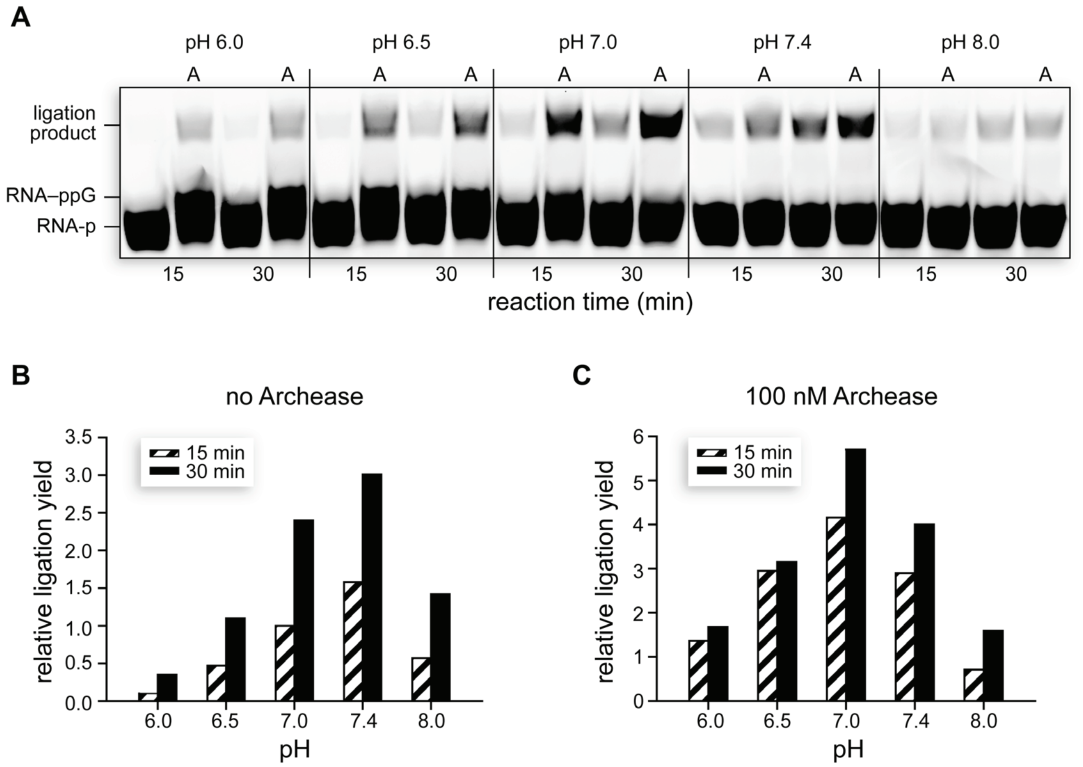

Figure S1. The pH dependence of RtcB catalyzed RNA ligation reactions. (**A**) Ligation reactions with RtcB alone (5 μM) or in the presence of Archease (100 nM, lanes labeled "A"). (**B**,**C**) Densitometry analysis of ligation reactions with the ligation yield plotted relative to that in the absence of Archease at 15 min. With RtcB alone, ligation was most efficient at pH 7.4. In the presence of Archease, however, ligation was most efficient at pH 7.0. Reaction mixtures contained RtcB (5 μM), GTP (100 μM), NaCl (300 mM), $MnCl_2$ (250 μM), and a 5′ and 3′ RNA fragment (1.0 μM each), and were incubated at 70 °C. Reactions at pH 6.0, 6.5, and 7.0 were in 50 mM Bis–Tris buffer, and reactions at pH 7.4 and 8.0 were in 50 mM Tris–HCl buffer. Buffers were adjusted to the appropriate pH at 70 °C.

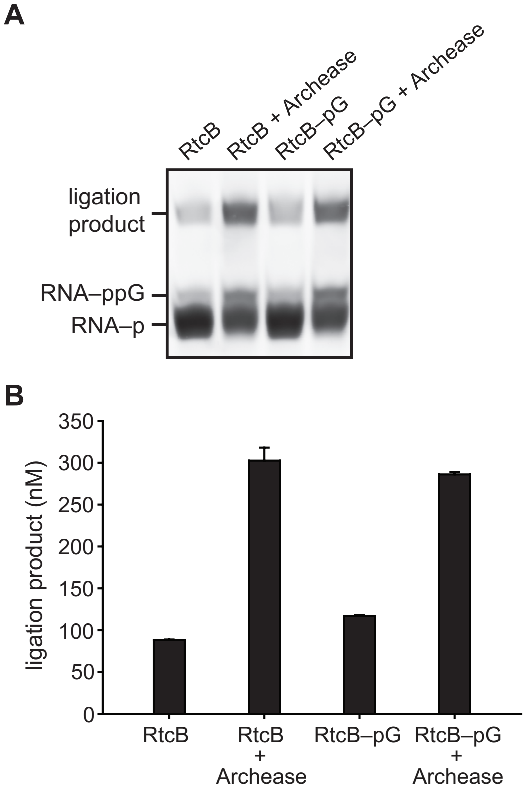

Figure S2. Ligation reactions testing the effects of RtcB pre-guanylylation. (**A**) RtcB was pre-guanylylated by incubation with GTP and Mn(II). Ligation reactions included 100 nM Archease where specified and were incubated at 70 °C for 30 min. (**B**) Graph of ligation product formed in reactions testing the effects of RtcB pre-guanylylation. The ligation product in the graph represents the average of two separate experiments ± SEM.

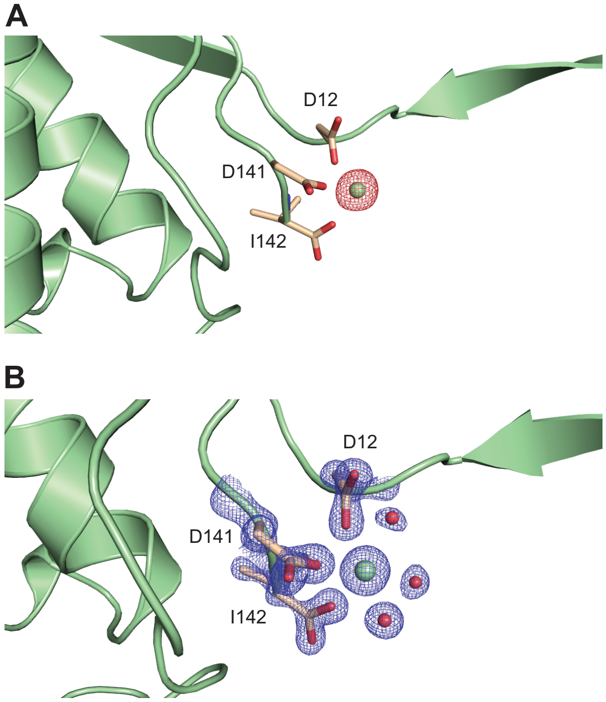

Figure S3. Electron density maps of the Archease subunit A metal-binding site. (**A**) Archease metal-binding site with the red mesh denoting an omit map ($F_o - F_c$) of the Ca(II) ion density contoured at 6σ. (**B**) Archease metal-binding site with the blue mesh denoting electron density ($2F_o - F_c$) of the refined model contoured at 2.6σ.

```
      H. sapiens  MKGGSRVSNPAVMAQEEEDVRDYNLTEEQKAIKAKYPPVNRKYEYLDHTA 50
 D. melanogaster  --------------MEVEFSRENFLLPEM------------KYEYLDHTA 24
      C. elegans  -------------MPSTSMIEDRSEIERR------------RFEYLDHPA 25
   P. horikoshii  ---------------------------------------MKKWEHYEHTA 11
     T. maritima  -----------------------------------------MRKPIEHTA 9

                  *
      H. sapiens  DVQLHAWGDTLEEAFEQCAMAMFGYMTDTGTVEPLQTVEVETQGDDLQSL 100
 D. melanogaster  DVQIHGWGSSLKEAFEQCGVAMFGYMTELDYVSVEQCFEIEAHGDDLESL 74
      C. elegans  DIQLHSWGSTMEEAFEACLVSMFGYMTDLAKVDEMYEFYWKASGDSLDGL 75
   P. horikoshii  DIGIRGYGDSLEEAFEAVAIALFDVMVNVNKVEKKEVREIEVEAEDLEAL 61
     T. maritima  DIAYEISGNSYEELLEEARNILLE---EEGIVLDTEEKEKMYPLEETEDA 56

      H. sapiens  LFHFLDEWLYKFSADEFFIPREVKVLSIDQRNFKLRSIGWGEEFSLSKHP 150
 D. melanogaster  LFHFLDELLFLFSAEPYLVCKKLEITKFDVENFEISCHCYGEPFELGKHP 124
      C. elegans  LFQFLDEALNSFHAEPCFVAKRVEILRFDKKKFEIEFRGWGESFDTSKHE 125
   P. horikoshii  LYSFLEELLVIHDIEGLVFRDFEVKIERVNGKYRLRAKAYGEKLDLKKHE 111
     T. maritima  FFDTVNDWILEISKG------------WAPWRIKREGNELKVTFRKIRKK 94

                                                **
      H. sapiens  QGTEVKAITYSAMQVYNE---ENPEVFVIIDI 179
 D. melanogaster  QGTEVKAITYSAMQIIQDVEASNYEVFVIIDI 156
      C. elegans  TEADIKSPTYSNMQINEK--PERCDIYVIVDI 155
   P. horikoshii  PKEEVKAITYHDMKIERLP-NGKWMAQLVPDI 142
     T. maritima  EGTEIKALTYHLLKFERDG--DVLKTKVVFDT 124
```

Figure S4. Sequence alignment of Archease proteins. The following species are included in the alignment (including NCBI accession numbers): *Homo sapiens* (AAN75223), *Drosophila melanogaster* (NP_650975), *Caenorhabditis elegans* (CCD62130), *Pyrococcus horikoshii* (O59205), and *Thermotoga maritima* (Q9X0H1). The alignment was generated with ClustalW2. Alanine-scanning substitutions that were tested in this study are highlighted in yellow. Residues coordinating a Ca(II) ion in the crystal structure of *P. horikoshii* Archease are denoted with an asterisk.

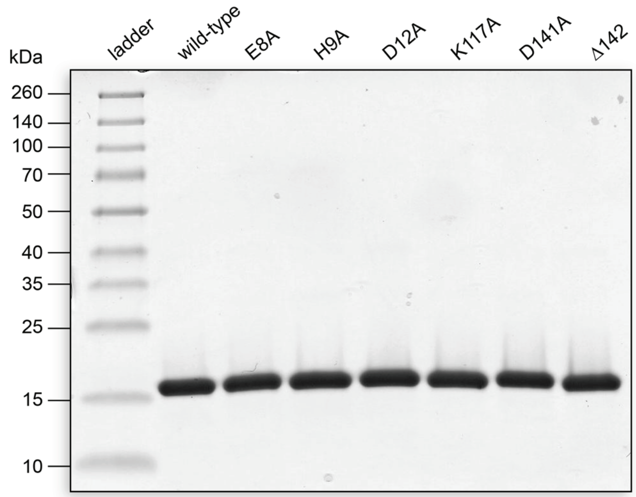

Figure S5. Coomassie-stained SDS–polyacrylamide gel showing the purity of Archease variants. Approximately 4 μg of an Archease variant was loaded in each lane. The identity of each variant is indicated at the *top*, and the molecular mass (kDa) is indicated on the *left*.

Table S1. Data collection and refinement statistics.

	P. horikoshii Archease
Space group	$P12_11$
Cell dimensions<br>*a*, *b*, *c* (Å)	72.22, 55.39, 87.09
Radiation Source	23-ID-D APS
Wavelength (Å)	0.9794
Resolution (Å)	43.95–1.44 (1.49–1.44)
Total Reflections	896897
Unique Reflections	118858 (9760)
R_{sym} or R_{merge}	0.075 (0.777)
R_{meas}	0.081 (0.836)
Mean $I/\sigma I$	13.42 (2.90)
Completeness (%)	94.50 (77.91)
Redundancy	7.5 (7.3)
R_{work} / R_{free}	0.143/0.179 (0.187/0.228)
CC1/2	0.96 (0.79)
CC*	1.00 (0.94)
B-factors (Å^2)	
Protein	14.7
Solvent	32.8
Ramachandran plot	98% favored
R.M.S. deviations	
Bond lengths (Å)	0.010
Bond angles (°)	1.25
Protomers	4
Protein residues	572
Heteroatoms	2 Ca(II) ions<br>2 MPD<br>4 acetate<br>886 waters
PDB ID	4n2p

Values in parentheses are for the highest-resolution shell.
